# Factors associated with maternal death in an intensive care unit

**DOI:** 10.5935/0103-507X.20160073

**Published:** 2016

**Authors:** Suzanne Vieira Saintrain, Juliana Gomes Ramalho de Oliveira, Maria Vieira de Lima Saintrain, Zenilda Vieira Bruno, Juliana Lima Nogueira Borges, Elizabeth De Francesco Daher, Geraldo Bezerra da Silva Jr

**Affiliations:** 1 Postgraduate Program in Public Health, Universidade de Fortaleza - Fortaleza (CE), Brazil.; 2 Maternidade Escola Assis Chateaubriand, Faculdade de Medicina, Universidade Federal do Ceará - Fortaleza (CE), Brazil.; 3 Department of Internal Medicine, Faculdade de Medicina, Universidade Federal do Ceará - Fortaleza (CE), Brazil.; 4 Medical School, Universidade de Fortaleza - Fortaleza (CE), Brazil.

**Keywords:** Maternal death/epidemiology, Women's health, Hypertension, pregnancy-induced, Risk factors, Intensive care units, Morte materna/epidemiologia, Saúde da mulher, Hipertensão induzida pela gravidez, Fatores de risco, Unidades de terapia intensiva

## Abstract

**Objective:**

To identify factors associated with maternal death in patients admitted to an intensive care unit.

**Methods:**

A cross-sectional study was conducted in a maternal intensive care unit. All medical records of patients admitted from January 2012 to December 2014 were reviewed. Pregnant and puerperal women were included; those with diagnoses of hydatidiform mole, ectopic pregnancy, or anembryonic pregnancy were excluded, as were patients admitted for non-obstetrical reasons. Death and hospital discharge were the outcomes subjected to comparative analysis.

**Results:**

A total of 373 patients aged 13 to 45 years were included. The causes for admission to the intensive care unit were hypertensive disorders of pregnancy, followed by heart disease, respiratory failure, and sepsis; complications included acute kidney injury (24.1%), hypotension (15.5%), bleeding (10.2%), and sepsis (6.7%). A total of 28 patients died (7.5%). Causes of death were hemorrhagic shock, multiple organ failure, respiratory failure, and sepsis. The independent risk factors associated with death were acute kidney injury (odds ratio [OR] = 6.77), hypotension (OR = 15.08), and respiratory failure (OR = 3.65).

**Conclusion:**

The frequency of deaths was low. Acute kidney injury, hypotension, and respiratory insufficiency were independent risk factors for maternal death.

## Abstract (pt)

**Objetivo:**

Identificar os fatores associados à morte materna em pacientes internadas em unidade de terapia intensiva.

**Métodos:**

Estudo do tipo transversal realizado em unidade de terapia intensiva materna. Foram selecionados todos os prontuários de pacientes admitidas no período de janeiro de 2012 a dezembro de 2014. O critério de inclusão foi todas as pacientes obstétricas e puérperas, e o de exclusão as com diagnóstico de mola hidatiforme, gravidez ectópica e anembrionada, e as internadas por causas não obstétricas. Foi realizada análise comparativa entre os desfechos óbito e alta hospitalar.

**Resultados:**

Foram incluídas 373 pacientes, com idade entre 13 a 45 anos. As causas de internação na unidade de terapia intensiva foram síndromes hipertensivas relacionadas à gestação, cardiopatias, insuficiência respiratória e sepse; as complicações foram lesão renal aguda (24,1%), hipotensão (15,5%), hemorragia (10,2%) e sepse (6,7%). Ocorreram 28 óbitos (7,5%). As causas de óbito foram choque hemorrágico, falência múltipla de órgãos, insuficiência respiratória e sepse. Os fatores de risco independentes para óbito foram lesão renal aguda (OR = 6,77), hipotensão (OR = 15,08) e insuficiência respiratória (OR = 3,65).

**Conclusão:**

A frequência de óbitos foi baixa. Lesão renal aguda, hipotensão e insuficiência respiratória foram os fatores de risco independentes associados à mortalidade materna.

## INTRODUCTION

The World Health Organization (WHO) defines maternal death (MD) as death occurring while pregnant or within 42 days of termination of pregnancy, from any cause related to pregnancy or its management, but not from accidental or incidental causes.^(1)^ MD is a serious public health problem, and it is evidence of the level of development of a given population.^(2)^ Approximately 99% of all MDs occur in developing countries.^(3-5)^

Most of these deaths could be avoided if health systems were to grant women access to high-quality services.^(2)^ In Brazil, the maternal mortality ratio was targeted to be less than or equal to 35 deaths per 100,000 live births by 2015,^(6)^ but this goal was not attained.^(7-9)^

Some studies have shown that, among pregnant women admitted to intensive care units (ICUs), the predominant diagnoses were conditions related to blood pressure disorders and that these women had not received adequate prenatal care.^(10)^ According to a WHO bulletin published in 2010, the incidence of admission to the ICU for pregnant Brazilian women was 2.1%.^(11)^ The main reasons for admission have been identified as hypertension syndromes, bleeding, and pregnancy-related sepsis.^(12-16)^

The aim of the present study was to identify factors related to maternal death among patients admitted to an intensive care unit.

## METHODS

The present cross-sectional study collected data from the medical records of patients admitted to the maternal ICU of *Maternidade Escola Assis Chateaubriand, Universidade Federal do Ceará*, Fortaleza, Ceará, which is a reference hospital in the region for gynecological, obstetrical, and neonatal care. Inaugurated in 1965, this maternity hospital is affiliated with the Brazilian Unified Health System (*Sistema Único de Saúde* - SUS) and supports 165 beds. The ICU has four beds for pregnant and puerperal women requiring advanced life support. The medical staff includes one attending physician and another on 24-hour call duty, both of whom have training in critical care; one obstetrician performs daily visits. In addition, there is one daytime nurse, who is the unit coordinator, one nurse per shift and two nurse technicians. The staff is completed by one physical therapist, who visits the patients daily.

All medical records of women admitted to the maternal ICU for pregnancy-related causes from January 2012 to December 2014 were reviewed. Pregnant and puerperal women were included, and those women with diagnoses of hydatidiform mole, ectopic pregnancy, or anembryonic pregnancy were excluded, as were those women admitted for non-pregnancy-related causes and cases with incomplete or non-existent records of the variables of interest. All medical records of the patients admitted to the study ICU were reviewed; all patients admitted for pregnancy-related causes were included. The study ICU also admits women with non-pregnancy-related problems-mostly gynecological disorders, such as complications from gynecological surgery-because this ICU is located at a school maternity hospital that is a referral center for the entire state of Ceará. All of the cases admitted to the ICU for pregnancy-related problems were included; the medical records of patients transferred to other hospitals were excluded from the analysis of risk factors for death because these patients were lost to follow up. Considering the number of live births in the state of Ceará during the study period (2012 to 2014: 380,425) and the maximum estimate of maternal mortality for the same period (88.6 per 100,000 live births), the sample size calculation produced an n = 114. A total of 465 admissions to the study ICU occurred during the study period. Of these, 92 patients were excluded, 85 because they did not meet the inclusion criteria and 7 because their medical records could not be located in the archive. Therefore, the sample consisted of 373 patients, a number which is representative of the targeted population.

The following variables were analyzed: age range, provenance (Fortaleza [the state capital] versus the interior of the state), provision of prenatal care, delivery type (vaginal or cesarean section), outcome (discharge, transfer, or death), and diagnosis upon admission to the ICU. Preexisting comorbidities, complications during the ICU stay, and interventions received (mechanical ventilation and pharmacological treatments) were also analyzed.

The study was assessed and approved by the Research Ethics Committee of the *Universidade de Fortaleza*, ruling no. 875,394. Being a retrospective study, informed consent was waived. A trustee form was signed that granted us access to the patients' medical records. An ad hoc form was filled per patient with the following data collected from the medical records: (1) identification - age, provenance, prenatal care and delivery type; (2) clinical history - diagnosis upon admission to the ICU, preexisting comorbidities, medications (vasoactive agents, antibiotics, and diuretics), and the use of mechanical ventilation; (3) clinical progression and complications (acute kidney injury - AKI was defined according to the Acute Kidney Injury Network - AKIN criteria),^(17)^ and (4) outcome (hospital discharge, transfer, or death).

The data were organized and processed using the Statistical Package for the Social Sciences (SPSS), version 20, and tabulated. The analysis of the results was based on analytical and descriptive statistics, using statistical tests to achieve the intended aims, especially the instrument's validity and reliability. P-values < 0.05 were considered statistically significant. Absolute and relative frequencies were calculated for all categorical variables. Mean, median, standard deviation, maximum, and minimum values were calculated for the numerical variables. The chi-square and Fisher exact tests were used to identify possible associations between covariates and main outcomes ("death" versus "hospital discharge"), and odds ratios (ORs) were used as measures of association. Next, a logistic model was fit to control for possible confounding factors and analyze causal relationships between outcomes (death or discharge) and exposure variables; variables with p-values ≤ 0.25 upon bivariate (association) analysis were included in the model. Comparative analysis was performed between the outcomes of death and hospital discharge to identify the main factors associated with MD.

## RESULTS

With respect to the sample's clinical and epidemiological characteristics, 68.1% of the patients were within the age range from 19 to 34 years; 51.7% came from Fortaleza; and 63% had received prenatal care. In regard to outcomes, death occurred in 7.6% of the sample. The clinical and epidemiological characteristics of the study sample are summarized in table 1. Table 2 describes the patients' diagnoses upon admission.

**Table 1:** Clinical and epidemiological characteristics of patients admitted to a maternal intensive care unit

Variables	N (%)
Age range (years)	
13 - 18	70 (18.8)
19 - 34	254 (68.1)
35 - 45	49 (13.1)
Provenance	
Fortaleza	193 (51.7)
Interior	180 (48.3)
Prenatal care	
No	13 (3.5)
Yes	235 (63)
Unknown	125 (33.5)
Delivery type	
Vaginal	43 (11.5)
Cesarean section	294 (78.8)
Outcome	
Discharge	345 (92.4)
Death	28 (7.6)

**Table 2:** Diagnosis upon admission of patients admitted to a maternal intensive care unit

Admission diagnosis*	N (%)
Hypertensive disorders of pregnancy	198 (53)
Eclampsia	69 (17.7)
Severe preeclampsia	66 (17)
HELLP syndrome	47 (12.1)
Pregnancy-induced hypertension	16 (4.1)
Hemorrhage/hemorrhagic shock	48 (12.3)
Heart disease	35 (9)
Respiratory failure	32 (8.2)
Sepsis	21 (5.4)
Placental abruption	14 (3.6)
Exogenous intoxication	5 (1.3)
Other	36 (9.3)

* Including patients transferred to other hospitals.

Comparative analysis between outcomes death and hospital discharge showed that 82.1% of the patients who died were within the age range of 19 to 34 years; 57.1% came from Fortaleza; and 60.7% of the medical records did not report whether prenatal care was received (Table 3). Approximately 39.9% of all patients exhibited some comorbidity; within the group who died, 35.7% exhibited some comorbidity versus 40.2% of those who were discharged.

**Table 3:** Outcomes of patients admitted to a maternal intensive care unit

Variables	Death N = 28 (%)	Discharge N = 345 (%)	OR (95%CI)	p value
Age range (years)				
13 - 18	4 (14.2)	66 (19.1)	2.8 (0.3 - 24.3)	0.173*
19 - 34	23 (82.1)	231 (66.9)	4.4 (0.6 - 32.0)	
35 - 45	1 (3.5)	48 (13.9)	1.0	
Provenance				
Fortaleza	16 (57.1)	177 (51.3)	1.2 (0.6 - 2.5)	0.566†
Interior	12 (42.8)	168 (48.6)	1.0	
Prenatal care				
No	1 (3.5)	12 (3.4)	1.8 (0.2 - 13.0)	0.008*
Unknown	17 (60.7)	108 (31.3)	3.2 (1.5 - 6.7)	
Yes	10 (35.7)	225 (65.2)	1.0	
Delivery type				
Vaginal	12 (42.8)	31 (8.9)	5.4 (2.7 - 10.8)	< 0.001†
Cesarean section	15 (53.5)	279 (80.8)	1.0	
Comorbidities				
Diabetes	1 (3.5)	11 (3.1)	1.1 (0.1 - 7.5)	1.000*
Hypertension	2 (7.1)	39 (11.3)	1.0	0.754*
Liver disease	4 (14.2)	5 (1.4)	6.7 (2.9 - 15.4)	0.003*
Coagulation disorders	4 (14.2)	7 (2.0)	5.4 (2.2 - 13.1)	0.005*
Complications during stay at ICU				
Acute kidney injury	19 (67.8)	71 (20.5)	6.6 (3.1 - 14.1)	< 0.001†
Oliguria	9 (32.1)	13 (3.7)	7.5 (3.8 - 14.7)	< 0.001*
Sepsis	6 (21.4)	19 (5.5)	3.8 (1.7 - 8.5)	0.010*
Hypotension	20 (71.4)	38 (11.0)	13,5 (6,2 - 29,3)	< 0.001*
Bleeding	8 (28.5)	30 (8.6)	3.5 (1.6 - 7.4)	0,004*
Respiratory failure	8 (28.5)	26 (7.5)	3,9 (1,9 - 8,3)	3.9 (1.9 - 8.3)
Use of mechanical ventilation				
No	-	269 (77.9)	-	< 0.001†
Yes	28 (100)	76 (22.0)	-	
Treatment				
Vasoactive drugs	26 (92.8)	41 (11.8)	59.3 (14.4 - 244.1)	< 0.001†
Antibióticos	21 (75)	146 (42.3)	3.7 (1.6 - 8.4)	0.001†
Diuretics	17 (60.7)	186 (53.9)	1.2 (0.6 - 2.6))	0.458†

ICU - intensive care unit.

* Fisher’s exact test;

† chi-square test.

The causes of death were as follows: hemorrhagic shock, 28.6%; multiple organ failure, 28.6%; respiratory failure, 28.6%; and sepsis, 14.3%. The multiple regression model evidenced that the factors associated with death (independent risk factors) were the following: AKI (OR = 6.77; 95% confidence interval [95%CI] = 2.6 - 17.4); hypotension (OR = 15.08; 95%CI = 5.8 - 38.8) and respiratory failure (OR = 3.65; 95%CI = 1.1 - 12.1).

## DISCUSSION

To analyze the factors associated with MD, an understanding of some individual characteristics of the patients admitted to the ICU is needed, as well as of the probable associations of these characteristics with the clinical outcomes. The sample of the present study mainly consisted of young women; the mortality rate did not differ significantly among the age ranges. These data are inconsistent with the results of other studies in which older maternal age was considered a risk factor for poorer prognosis.^(18)^

The proportion of patients from the capital and interior of the state was similar, which shows that even patients from large urban centers and thus with (theoretically) greater access to healthcare services might nevertheless have poor clinical progressions due to disorders of avoidable cause. The fact that in 33.5% of the medical records there was no mention of prenatal care, which hindered the reach and efficiency of analysis, is noteworthy. As is already known, prenatal care still poses a challenge to the Brazilian SUS. One study has shown that the coverage of prenatal care in Brazil is practically universal (98.7%), yet it is not yet adequately carried out due to its delayed adoption and limited number of visits. That same study found that the main reasons for women from the Northern and Northeastern regions and for those with low educational levels to forego prenatal care were barriers to access,^(19)^ which may be understood as gaps in the care provided in these two large Brazilian areas, as well as a result of limitations derived from poor education. Another study also conducted in the Northeastern region that assessed factors associated to maternal "near misses"-i.e., conditions with high mortality-found that more than half of the sample had not received adequate prenatal care, with 4.9% of the patients having had no visits, and 49.2% having had fewer than six visits.^(20)^

According to the "*Nascer no Brasil* study" ("Born in Brazil study"), conducted in 2012, 60% of pregnant women started prenatal care late, i.e., after gestational week 12, and approximately one quarter of them attended fewer than the minimum six visits recommended by the Brazilian Health Ministry.^(21)^ The reasons for non-adherence to prenatal care in Brazil should be investigated at the primary care level to reinforce efforts toward the active search for and follow up of pregnant women. Some studies have implicated inadequate prenatal care as an independent risk factor for MD.^(22)^

Cesarean section was the most frequent type of delivery performed over the study period (78.8%), which is somewhat expected in the case of women with complications admitted to the hospital and who frequently meet the indications for pregnancy termination, especially those with hypertensive syndromes. This finding agrees with the findings of other studies.^(10,14)^^)^ Within the clinical context of patients, and more particularly in cases of high-risk pregnancy, cesarean section might be advantageous in that it reduces perinatal morbidity and mortality. Nevertheless, this procedure exposes women to higher risk of puerperal infection, bleeding, and anesthesia accidents.^(9)^

The sample recruited for the present study consisted of women admitted to the ICU; consequently, the analysis of risk factors for death concerned a population of severe patients with potentially fatal complications during delivery and the puerperal period. The death rate was 7.6%, a rate similar to those reported in other studies.^(10)^^)^ ICU mortality reported in the literature varies from 5% to 30%.^(11)^ The low death rate found in the present study is a reflection of the high quality of care delivered upon admission to and during the stay at the investigated ICU.

The main diagnoses determining admission to the ICU were hypertensive disorders of pregnancy (eclampsia, severe preeclampsia, HELLP syndrome [Hemolysis, Elevated Liver enzymes, Low Platelet count], and pregnancy-induced hypertension). Hemorrhagic shock, heart disease, respiratory failure, and sepsis were also frequent reasons for admission. These findings corroborate previous reports in the literature.^(10,23,24)^^)^ Eclampsia and severe preeclampsia accounted for 34.7% of admissions. One study found that in reference hospitals for high-risk pregnancies, preeclampsia/eclampsia, urinary tract infection, and puerperal infection predominate among the direct pregnancy-related causes of death.^(4)^ Therefore, the quality of the care provided might be a decisive factor.

In developed countries, the main cause of admission of pregnant women to the ICU is preexisting or acquired heart disease.^(25)^ This difference might be due to socioeconomic conditions, the quality of prenatal care, or better modalities of treatment in the ICU.^(26)^

Comparative analysis between the death and discharge outcomes is crucial considering the current relevance of the concept of "maternal near miss"-namely, the circumstance in which women who develop severe complications during pregnancy, delivery, or the puerperal period survive.^(15)^ The reason for such emphasis is that comparative analysis might reveal characteristics decisive for both outcomes and guide early interventions to reduce damage to maternal health.

The fact that 65.2% of the women who were discharged had received prenatal care raises questions about the quality of the care actually received. One study conducted at two municipalities in the Northeastern region of Brazil found that one out of five women received inadequate prenatal care (defining "adequate" as prenatal care a first visit by gestation day 120, with at least six visits in total), a prevalence considered high.^(27)^ Because many of the actions are scheduled according to the period of pregnancy, a small number of visits might compromise the quality of prenatal care because the opportunities for early intervention are reduced.

Regarding delivery type, the proportion of vaginal births and cesarean sections was similar among the patients who died, thus differing from the women who were discharged. This study found that a large number of patients had hemorrhagic disorders and that bleeding was one of the factors related to death; consequently, vaginal delivery was associated with higher mortality compared to cesarean section. In this sample, vaginal delivery was significantly associated with MD (5.47-fold higher risk of death). One study has indicated that women subjected to labor induction followed by vaginal delivery are at higher risk of bleeding.^(28)^ Additionally, hemorrhage is the main cause of MD in the United Stated, and two-thirds of births in the country are via vaginal delivery.^(28)^ Furthermore, bleeding disorders in association with hypotension can lead to kidney dysfunction,^(29)^ which explains the occurrence of AKI, a relevant risk factor for death, in a considerable fraction of the patients included in the present study.

Overall, the absence versus presence of comorbidities did not have a statistically significant effect. However, the higher prevalence of comorbidities among the women who died was attributable to liver disease and coagulation disorders-both of which are conditions related to HELLP syndrome. The risks of death for women with liver disease and for coagulation disorders were almost 7 and 5.48 times higher, respectively, compared to the women with other comorbidities, such as diabetes, hypertension, and heart disease, among others. HELLP syndrome is a severe complication with high morbidity and mortality, and it affects 4% to 12% of women with preeclampsia or eclampsia.^(9)^

In a study conducted in the United Kingdom, comorbidities were significantly more common among the patients who died,^(23)^ a finding opposite that of the present study: of the patients who died, 35.7% exhibited some comorbidity during pregnancy versus 40.2% among those who were discharged. These data once again point to the regional and care-related peculiarities of MD.

The association of comorbidities with other factors, such as socioeconomic status and low educational level, revealed that comorbidities worsen maternal state of health. Some studies have shown that these factors are directly related to MD by influencing self-care, interfering with the quality of prenatal care, and attenuating the effectiveness of treatment.^(14,17,26)^

Among the main complications developed during a stay at the ICU, AKI stands out, having affected 24% of the total sample and 67.8% of the patients who died. AKI is even more frequently used in clinical ICUs, reaching up to 40% of cases, and it is associated with higher mortality.^(30)^ In a study by Silva Junior et al.^(31)^ with patients receiving care at the obstetrical clinic of a referral hospital in Fortaleza, severe AKI (requiring dialysis) occurred in less than 1% of the sample. This finding shows that this diagnosis is mainly related to more severe cases-in the present study, AKI, in all its stages, stood out due to its high incidence. The authors of a previous study also found that the factors associated with death were hypotension (OR = 43.7) and hyperbilirubinemia (OR = 1.22), whereas bicarbonate > 20mEq/L was protective against death.^(31)^ In the present study, AKI was significantly associated with death upon inclusion in the multivariate analysis.

AKI is a frequent and potentially fatal complication among pregnant women in developing countries, its cause being predominantly related to hypertensive disorders.^(32)^ The main risk factors for the development of AKI in the ICU are the following: age above 55 years; Acute Physiology and Chronic Health Evaluation (APACHE) II score > 16; baseline creatinine > 1.2 mg/dL; previous use of nonsteroidal anti-inflammatory drugs; and septic shock.^(33)^

Sepsis, which in the present study was a justification for admission to the ICU and an explanation for complications and death, is defined as a systemic inflammatory response syndrome secondary to infection.^(34)^ Pregnancy predisposes women to four specific infectious complications: pyelonephritis, chorioamnionitis (including septic abortion), endometritis (often developing after cesarean section), and pneumonia. Most infections appear after delivery, for which the main risk factor is cesarean section. Clinical manifestations of sepsis include signs of systemic inflammation, which might be followed by coagulation disorders, vasoplegia, and eventual multiple organ failure.^(35)^

Significant numbers of obstetrical patients with infectious complications are still a striking aspect of the profile of MD in Brazil, as several studies conducted in various regions have shown.^(10,15,16)^ This phenomenon might be related to the alarming number of cesarean sections performed annually. In the *Nascer no Brasil* study, approximately 52% of births were by cesarean section, and it is estimated that about one million women undergo this surgery without any consistent obstetrical reason, exposing them to higher risks of complications and death.^(22)^

The data on the use of mechanical ventilation and pharmacological treatment received by the patients in the sample are directly related to the complications developed by these patients; such data are useful in confirming both the severity of the analyzed cases and the efficacy of treatment, as the number of deaths was low. It is worth observing that 100% of the women who died had been subjected to mechanical ventilation, whereas 77.9% of the discharged ones had been not. Avoidance of mechanical ventilation might be considered a protective factor related to lower exposure to infectious agents.

The main causes of death found in the present study were as follows: hemorrhagic shock, multiple organ failure, respiratory failure, and sepsis. In a study conducted at a referral center in southeastern Brazil, the most frequent causes of MD were infection, hypertensive disorders, and bleeding;^(15)^ these causes were similar to those reported in a study conducted in the southern region: cardiogenic shock, HELLP syndrome and hemorrhagic shock.^(10)^

However, upon multivariate/logistic regression analysis, the factors independently associated with death were AKI, hypotension, and respiratory failure, with all three related to hypertensive disorders of pregnancy, as well as infectious complications progressing into sepsis; most of these deaths are considered to be avoidable. Ponce et al.^(33)^ found that AKI is independently associated with longer stays at the ICU and high mortality.

The present study has some limitations. First, the accuracy of the collected data may be questioned, as medical records do not always contain accurate information (a limitation inherent to retrospective studies). Lack of information on income, race/skin color, and educational level, for instance, is a gap that cannot be overlooked, due to the relevance of these factors for a pregnant women's understanding of the healthcare instructions provided during prenatal care visits. Second, the fact that the study sample consisted of patients receiving care at a single maternity hospital restricts the results of the present study, which thus precludes generalization. However, as the study setting was a large hospital that is a referral center for the entire state of Ceará, the results could be expected to be valid for other locations and might contribute to the reduction of maternal mortality.

## CONCLUSION

Several different factors may be associated with maternal death among women admitted to the intensive care unit. Hypertensive syndromes were the main reason for admission, as the patients experienced complications such as acute kidney injury, hypotension/bleeding, and sepsis. These findings evidence the fragility of prenatal care in the targeted Brazilian region, as most cases of pregnancy-induced hypertension can be prevented via adequate prenatal care.

The frequency of deaths was low despite the patients' severity. This finding reflects the high quality of the in-hospital obstetrical care delivered based on adequate technical and structural support. It is expected that the results obtained will contribute to increasing professionals' knowledge of the factors associated with maternal death, ground the planning of strategies and programs of prevention against such factors (particularly reinforcing the relevance of careful prenatal care), and encourage further studies based on the assumptions raised.
